# Sex Differences in Antidepressant Effect of Sertraline in Transgenic Mouse Models

**DOI:** 10.3389/fncel.2019.00024

**Published:** 2019-02-01

**Authors:** Lei Ma, Yong Xu, Wei Jiang, Yuhong Li, Xinzhu Zhang, Gang Wang, Rena Li

**Affiliations:** ^1^The National Clinical Research Center for Mental Disorders and Beijing Key Laboratory of Mental Disorders, Beijing Anding Hospital, Capital Medical University, Beijing, China; ^2^Advanced Innovation Center for Human Brain Protection, Capital Medical University, Beijing, China; ^3^Qingdao Municipal Hospital, Qingdao, China; ^4^School of Life Sciences, University of Science and Technology of China, Hefei, China; ^5^Center for Hormone Advanced Science and Education, Roskamp Institute, Sarasota, FL, United States; ^6^Beijing Institute for Brain Disorders, Capital Medical University, Beijing, China

**Keywords:** sex difference, sertraline, 5-HT, DA, estrogen

## Abstract

The main purpose of this study is to explore sex differences in the antidepressant effect of sertraline in genetic knockout or overexpression estrogen-synthesizing enzyme aromatase (Ar) gene mouse models in the forced swim test (FST). Our results demonstrated a significant reduction of depression-like behavior in the mice with overexpression of brain aromatase (Thy1-Ar) compared to sex- and age-matched Ar^+/−^ mice or wild type control mice. Using HPLC analysis, we also found an association between the brain estrogen-related antidepressive behavior and the regulation of serotonin (5-HT) system. Interestingly, a single dose administration of sertraline (10 mg/kg, i.p.) induced reduction of immobility time was found in all genotypes, except male Ar^+/−^ mice. While the underlying mechanisms of sex-specific response on antidepressive effect of sertraline remain to be investigated, our data showed that female mice appear to be more sensitive to sertraline-induced changes of 5-HT system than male mice in the prefrontal cortex (PFC) and the hippocampus (HPC). Further investigation of sex-specific effect of brain estrogen on antidepressant is needed.

## Introduction

The prevalence of major depressive disorder (MDD) is more common in women than men (Pearson et al., 2013). Sex hormones may play an important role in this gender difference as increased likelihood of depression in menopausal women as well as postpartum depression occurs soon after giving birth (Soares, 2014). While lower estrogen levels have been specifically implicated in this increased risk in post-menopausal women (Albert et al., 2015), the hypothesis of sex hormone-related depression is also supported by animal studies, such as more depression-like behaviors were seen in the ovariectomized (OVX) rodents vs. intact rodents (Ye et al., 2016). Furthermore, similar to the human clinical practice of treating depression with estrogen (Toffol et al., 2015), many animal studies suggest that estrogen supplementation can prolong the swimming time in the forced swim test (FST) as a support of estrogen antidepressive effect (Walf and Frye, 2010a; Saravi et al., 2016).

In addition to sex difference in depression, interestingly, clinical studies showed that women with depression may respond better to serotonin reuptake inhibitors (SRIs) than depressive men (Baca et al., 2004; Berlanga and Flores-Ramos, 2006). Similar sex differences in SRIs response are also seen in animal tests (Fernandez-Guasti et al., 2017). While very little is known about the mechanisms of this sex-dependent differential response, we hypothesized that estrogen level might be responsible for the sex-dependent SRIs action.

Aromatase is the rate-limiting enzyme that catalyzes the conversion of androstenedione or testosterone to estrogens (Di Nardo and Gilardi, 2013). The level of estrogen in the brain depends largely on the expression of aromatase (Prange-Kiel and Rune, 2006). Brain-derived estrogen has a greater impact on brain function and neuronal diseases than the estrogen in circulation (Yue et al., 2005). This suggests that investigation of brain aromatase may clarify the mechanism of differences in gender response.

In the current study, we investigated gender differences to SRI response with a genetic mouse model of estrogen deficiency and a transgenic mouse mode of brain estrogen overexpression. We first examined the antidepressive effect of sertraline, one of classic SRIs, in behavioral test such as immobility time in the FST. Then we investigated brain monoamine system such as serotonin (5-HT), dopamine (DA), and their metabolites in the prefrontal cortex (PFC) and the hippocampus (HPC) of the experimental mice. Lastly, we analyzed the association between sex hormone levels and the antidepressive effect of sertraline in both male and female transgenic mice.

## Materials and Methods

### Animals

Aromatase-knockout (Ar^−/−^) mice (background: C57Bl/6Jmice) were generated by deleting exons1 and 2 encoded by the CYP19 gene as described (Honda et al., 1998). Heterozygous males and females were generated when breeding a homozygous-null male mouse to a wild type (WT) female mouse. Neuron-specific aromatase expression mice (Thy1-Ar) were generated by modifying marine thy1.2 genomic expression cassette for driving human aromatase expression in the nervous system. All mice (8–12 weeks old) were maintained in groups-housed four per cage, a 12 h light-dark cycle (lights on at 0800), and kept at a constant 24 ± 1°C temperature. Food and drinking water were unlimited. Mice were randomly divided into sertraline treatment group and vehicle treatment group. Each experimental group consisted of 6–7 mice. All animal studies and experimental procedures were in conformity to National Institutes of Health Guide for the Care and Use of Laboratory Animals and approved by the Ethical Committee for Animal Use, University of Science and Technology of China.

### Genotyping

Genomic DNA extraction from toe tissue was performed by incubating samples in NaOH (50 mM) at 99°C for 30 min. Samples were centrifuged and Tris-HCL (1M; PH 7.4) was added. One μl of the supernatant was used for PCR amplification. Primers specific for the Ar^+/−^ mice (sense: 5′-CTTGT CTAAG TGTCC AATCAC-3′; antisense: 5′-TTACC ATGTC CTAAT CTTCAC-3′), and primers specific for Thy1-Ar mice (sense: 5′-AGCCC TCAAG GTAAA TGGGGA-3; antisense: 5′-GAGGA TGTGC CCTCA TAATTCC-3′) were used. After initial denaturation at 94°C for 5 min, reactions were subjected to 35 cycles of 94, 60, and 72°C for 30 s each. PCR products were assessed by electrophoresis on agarose gel with concentration of 2% and detected by gel imaging analysis system (CLINX, Shanghai).

### Drug Treatment

Mice received a single injection intraperitoneally (i.p.) of distilled water (10 ml/kg) or sertraline hydrochloride (Sigma, United States, dissolved in distilled water) (1 mg/ml), in a volume of 10 ml/kg of body weight. The dose was chosen on the basis of previous studies (Mouri et al., 2012).

### Forced Swim Test (FST)

The FSTs were conducted in keeping with previous method with minor modifications (Autry et al., 2011). The experimental mice were placed in the behavioral test room for 2 h before the FST. Individual mice were placed in a cylindrical tank (25 cm tall × 14 cm diameter) containing15 cm of water with temperature at 24 ± 2°C. Thirty minutes after injection, mice were videotaped in the FST for 6 min. During the last 4 min, floating and remaining motionless were defined as the immobile and the duration was recorded. In order to minimize circadian influence, FSTs were consistently performed between 2100 and 2400 h. Mice were sacrificed by decapitation immediately after FST. The HPC and the PFC of mice were dissected on an ice-cold plate and stored at −80°Cfor later tissue analysis.

### Spontaneous Locomotor Activity

Prior to FST, each animal was put into the center of the open-field square (50 cm × 50 cm × 50 cm) 30 min after administration with sertraline or vehicle. Between each mouse exposure the apparatus was cleaned with 10% ethanol and rinsed with hot water twice. The distance traveled (cm) was recorded for 5 min using a digital video-camera. A video tracking software EthoVision XT (Noldus, Netherlands) was used to analyze the horizontal locomotor activity of each mouse.

### High-Performance Liquid Chromatography (HPLC)

Brain homogenates underwent a deproteinization step with 0.2 mL ice-cold perchloric acid (0.4 M) and then centrifugation for 20 min (12,000 × g, 4°C). Twenty μl clear supernatant were filtered by 0.22 mm Cellulose filters (Millipore, United States). The resulting sample was injected into the HPLC system with electrochemical detection (Model 5600A; Coularray Detector System, ESA, Chelmsford, MA, United States). The analytic results consisted of DA, 3,4-Dihydroxyphenylacetic acid (DOPAC), 5-HT, 5-Hydroxyindoleacetic acid (5-HIAA) and homovanillic acid (HVA), were expressed as μg/g protein to present. The ratios of DOPAC plus HVA to DA and 5-HIAA to 5-HT are an index of DA and 5-HT turnover rate, respectively (Santos et al., 2010).

### Statistical Analysis

Data were evaluated using SPSSv.20 statistical software (IBM; United States). Two-way analysis of variance (ANOVA) considering genotype as one factor and treatment (vehicle, sertraline) as the other factor followed by Bonferroni *post hoc* test was performed for depression-like behaviors and neurotransmitters. All data were presented as means ± SEM and *P* < 0.05 were deemed statistical significance.

## Results

### Enriched Brain Estrogen Synthesis Expressed Antidepressive-Like Behavior in Male and Female Thy1-Ar Mice During the FST

**FIGURE 1:**
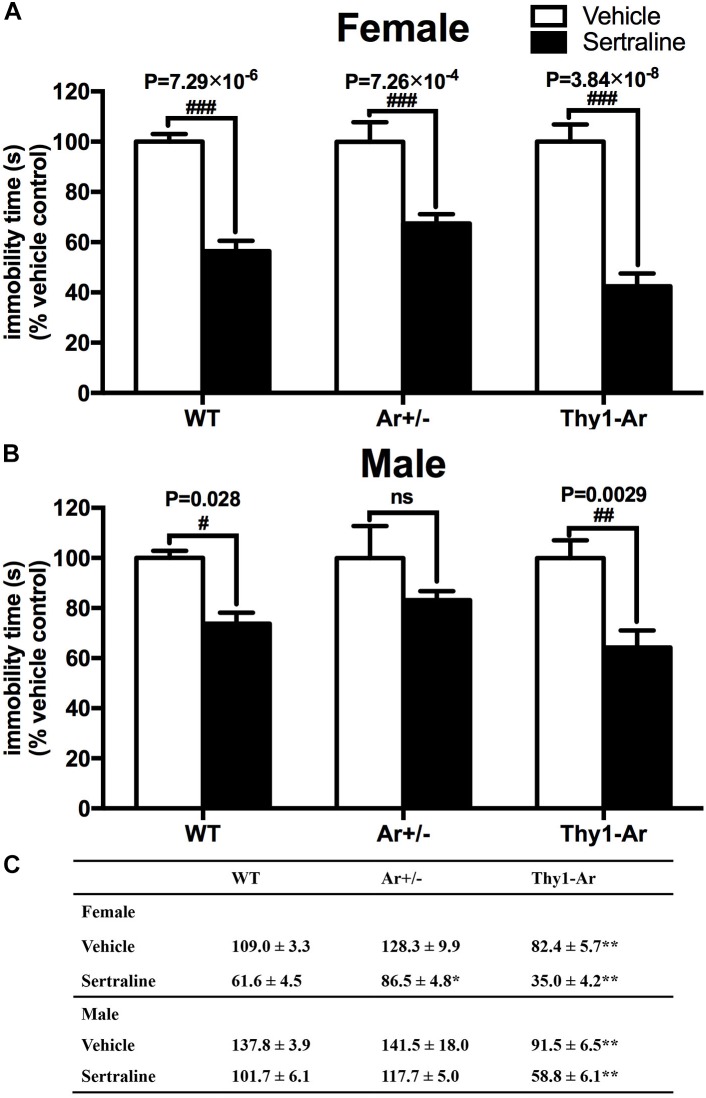
Enriched brain estrogen synthesis expressed antidepressive-like behavior and promoted antidepressive effect of sertraline in both male and female mice. Effect of sertraline on percentage change of immobility time during the FST from vehicle treated mice in both **(A)** females and **(B)** males. Mean immobility duration **(C)** in sec. of female and male mice after sertraline administration or vehicle during the FST. Data represent means ± SEM (*N* = 6–7 mice/group), as evidenced by two-way ANOVA. ^#^*P* < 0.05, ^##^*P* < 0.01, ^###^*P* < 0.001 versus vehicle-treated group; ^∗^*P* < 0.05, ^∗∗^*P* < 0.01 versus WT mice.

Mice with three genotypes as WT, Ar^+/−^ and Thy1-Ar were used to study the effect of endogenous estrogen on depression-like behaviors. As shown in Figure 1C, vehicle-treated Thy1-Ar mice showed a significant less immobility time (∼30% in females, ∼40% in males) compared to sex-matched WT mice. There is no difference in immobility time between male and female mice in all three genotypes. Unexpectedly, we found no effect of endogenous estrogen deficiency on immobile time in the FST in both male and female Ar^+/−^ mice compared to sex-matched WT animals. These results imply that enriched brain estrogen promotes antidepressive-like behavior, whether reduction of endogenous estrogen failed to alter immobility time during the FST.

### Enriched Brain Estrogen Promotes Antidepressive Effect of Sertraline in Both Male and Female Mice

Sertraline significantly reduced the immobility time during FST in all female animals when compared with their vehicle treated counterparts (Figure 1A). Sertraline administration induced much greater reduction of immobility time in female Thy1-Ar mice (*P* = 3.8 × 10^−8^) while female Ar^+/−^ mice showed less reduction of immobility time (*P* = 7.3 × 10^−4^) compared to that in sex-matched WT mice (*P* = 7.3 × 10^−6^). Interestingly, male animals responded to the sertraline administration differently. First, while both male and female Thy1-Ar mice responded to sertraline-induced antidepressive effect in immobility time, male Thy1-Ar mice showed less sensitive to sertraline treatment than female Thy1-Ar mice (*P* = 0.0029 vs. *P* = 0.000000038) compared to sex-matched WT mice. In addition, we found sertraline administration induced no significant reduction of depressive-like behavior in male Ar^+/−^ mice (Figure 1B), while female Ar^+/−^ mice had significant but less response to sertraline than sex-matched WT and Thy1-Ar mice (Figure 1A).

### Sertraline Did Not Alter Spontaneous Locomotor Activity in All Three Genotypes Regardless of Sex Difference

**FIGURE 2:**
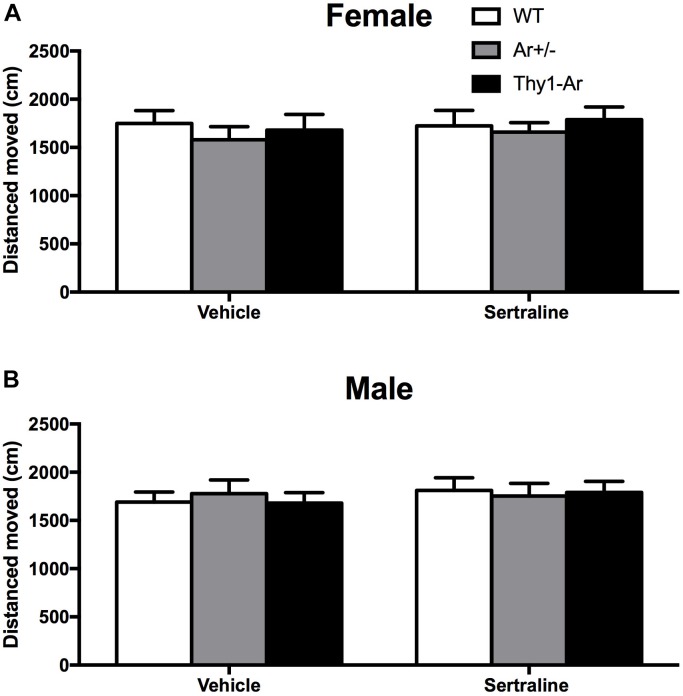
Sertraline did not alter spontaneous locomotor activity in all three genotypes regardless of sex difference. No differences were found in total distance moved in spontaneous locomotor activity in both **(A)** female and **(B)** male mice treated by vehicle or sertraline. Data represent means ± SEM (*N* = 6–7 mice/group), as evidenced by the two-way ANOVA.

To examine whether the endogenous estrogen-related behaviors is depressive specific, we also included open field test for locomotor activity in all three genotypes mice. As shown in Figure 2, there were no differences in distance moved in the open field behavioral test among the WT, Ar^+/−^, Thy1-Ar mice regardless sexes. In addition, sertraline treatment did not alter the spontaneous locomotor activity in all of the experimental mice. Our data suggested that endogenous estrogen induced no significant drug-effect or sex-effect on spontaneous locomotion.

### Endogenous Estrogen Induced Sex- and Brain Region-Specific Alterations in 5-TH Systems

**FIGURE 3:**
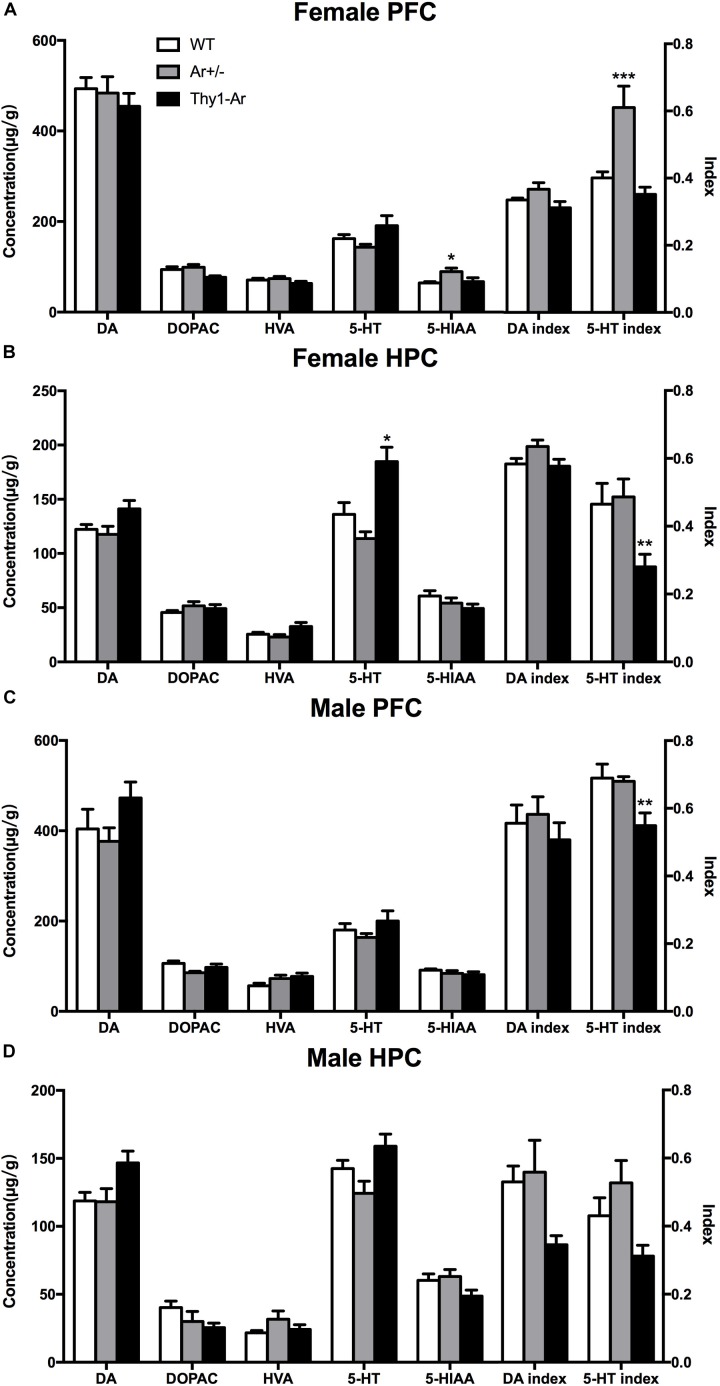
Endogenous estrogen induced sex- and brain region-specific alterations in 5-TH systems. The concentration (μg/g) of DA and 5-TH related neurotransmitters affected by endogenous estrogen in **(A)** female PFC, **(B)** female HPC, **(C)** male PFC, and **(D)** male HPC. Data represent means ± SEM (*N* = 6–7 mice/group), as evidenced by the two-way ANOVA followed by Bonferroni *post hoc* test. ^∗^*P* < 0.05, ^∗∗^*P* < 0.01, ^∗∗∗^*P* < 0.001 versus WT mice.

To further understand the role of endogenous estrogen in depressive behaviors, we also measured neurotransmitters DA and 5-HT and their metabolites in the HPC and the PFC of mice. As shown in Figure 3, we found no significant effect of endogenous estrogen on DA, DOPAC, HVA, and DA index in regardless sexes and brain regions. For the 5-HT system, our data showed an elevated level of 5-HIAA and 5-HT turnover rate without the change of 5-HT in the PFC of female Ar^+/−^ mice compared to that of WT females (Figure 3A), while a higher 5-HT level and lower 5-HT turnover rate were observed in the HPC of female Thy1-Ar mice (Figure 3B). On the other hand, no differences were detected in 5-HT or 5HIAA levels in all male genotypes regardless brain regions (Figures 3C,D), except a significantly decreased 5-HT index was observed in the PFC of male Thy1-Ar mice compared to male WT mice.

### Sex- and Brain Region-Specific Differences in DA and 5-TH Systems Following Sertraline Administration

**FIGURE 4:**
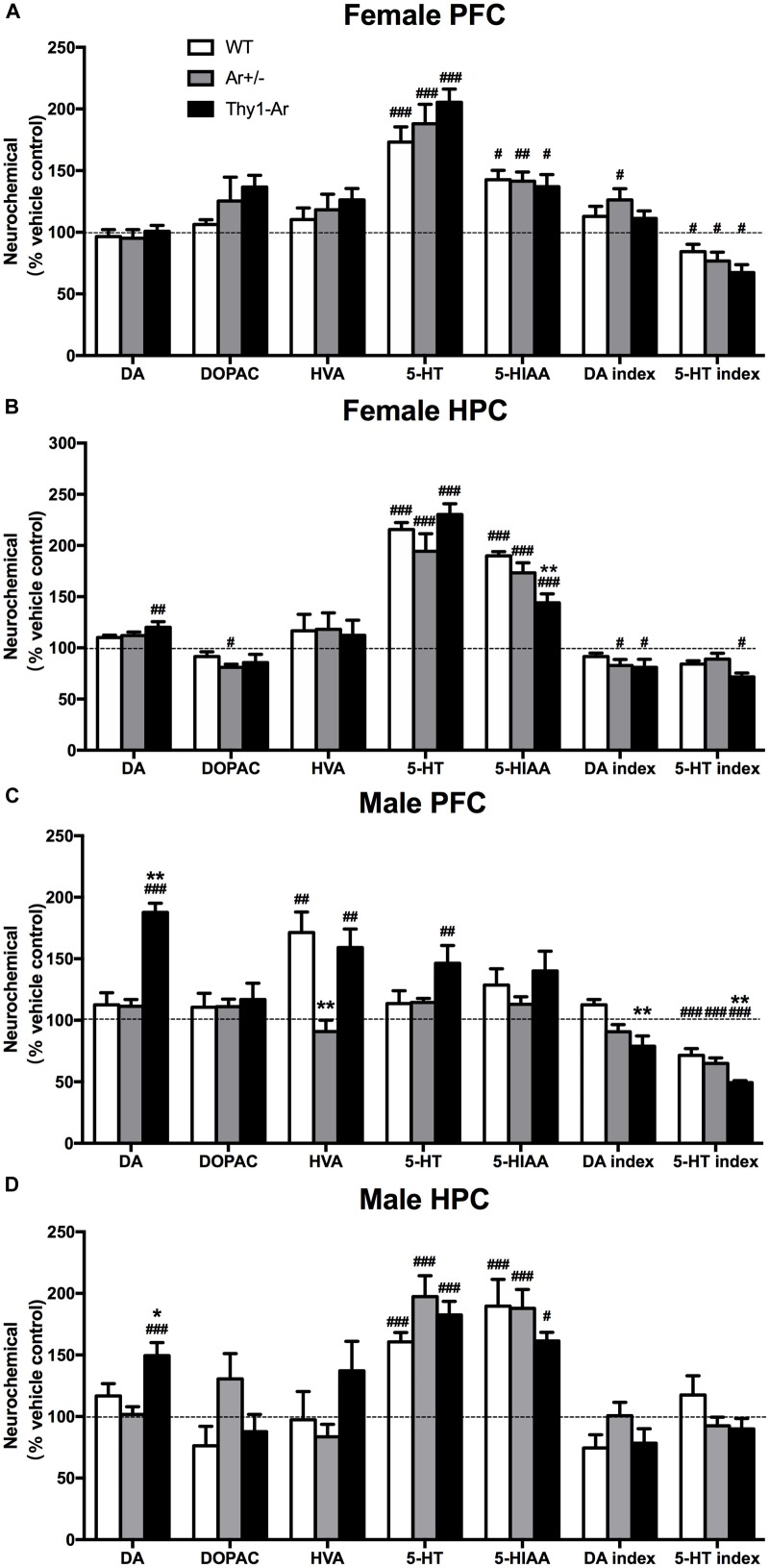
Sex- and brain region- specific differences in DA and 5-TH systems following sertraline administration. The percentage of DA and 5-TH related neurotransmitters for sertraline-treated and vehicle-treated in **(A)** female PFC, **(B)** female HPC, **(C)** male PFC, and **(D)** male HPC. Data represent means ± SEM (*N* = 6–7 mice/group), as evidenced by the two-way ANOVA followed by Bonferroni *post hoc* test. ^#^*P* < 0.05, ^##^*P* < 0.01, ^###^*P* < 0.001 versus vehicle group; ^∗^*P* < 0.05, ^∗∗^*P* < 0.01 versus WT mice.

To investigate changes of neurotransmitters induced by SRI in males and females, relevant values of DA and 5-HT system were measured by HPLC analyses after sertraline administration. For the DA system, sertraline showed no effect on DA level and its metabolites in the PFC of all three genotype female mice (Figure 4A), while sertraline showed a mild elevation of DA level and reduction of DA index in the HPC of female Thy1-Ar mice, and a marginally significant reduction of DOPAC and DA index levels in the HPC of female Ar^+/−^ mice as compared to the vehicle-treated genotype-matched female mice (Figure 4B). In males, sertraline was associated with elevated DA levels only in both brain regions of Thy1-Ar mice, while the enhanced HVA levels were found only in the PFC of WT and Thy1-Ar males (Figures 4C,D). Meanwhile, male Thy1-Ar mice responded better to sertraline treatment implemented by increasing DA level in both areas and decreasing DA index only in the PFC as compared with male WT mice. It is worth to note that male mice with estrogen deficiency failed to respond to sertraline treatment in DA relevant values (Figures 4C,D). For the 5-HT system, our data showed that sertraline administration also significantly promoted the levels of 5-HT and 5-HIAA from female animals regardless of genotypes. Decreased ratio of 5-HIAA/5-HT induced by sertraline was observed in both PFC and HPC of female Thy1-Ar mice, while such result was observed only in the PFC of WT and Ar^+/−^ females (Figures 4A,B). Interestingly, the sertraline-induced elevation of 5-HT and 5-HIAA levels were only obtained in the HPC of male mice regardless of genotypes, while no changes were found in the PFC except in male Thy1-Ar mice (Figures 4C,D).

## Discussion

In this study, we used transgenic mouse models to examine the effect of estrogen on sertraline-induced behavioral and biochemical changes. First, we demonstrated that an elevation of brain estrogen level in Thy1-Ar mice (Supplementary Figure S1) exhibited less depression-like behaviors in the FST than sex-matched WT mice (Figure 1). This estrogen enriched antidepressive-like behaviors were consistent with reports from other group which demonstrated that increased estrogen levels by estrogen supplementation could reverse depression-like behavior in ovariectomized rodents (Walf and Frye, 2010b). Instead of giving estrogen treatment, our study used the newly developed brain-specific aromatase transgenic mice, Thy1-Ar, reported that elevation of brain estrogen level can reduce depression-like behavior in both sexes. However, the Ar^+/−^ mice, a model of estrogen deficiency as we previous published (Yue et al., 2005; McAllister et al., 2010), showed no changes in immobility time compared to sex-matched WT mice (Figure 1). There are several possible explanations for the weak effect of Ar^+/−^ mice on depressive behaviors. For example, we think the level of estrogen reduction is critical for depressive-like behavior. While our data showed that increase brain estrogen (Thy1-Ar female mice) had less depression-like behavior, other report demonstrated a significant increase in depression-like behavior in homozygous aromatase knockout (Ar^−/−^) female mice compared to WT (Dalla et al., 2004). However, how essential does the level of endogenous estrogen plays in depression is unclear since studies demonstrated estrogen level-related mood and behaviors changes during the menstrual cycle in reproductive women (Jang and Elfenbein, 2018), as well as in female animals (Estrada-Camarena et al., 2011). As the changes of endogenous estrogen level in Ar^+/−^ and Thy1-Ar mice are much greater than that occurred in regular estrous cycles for WT mice, we hypothesize that the behaviors changes in FST from these aromatase animal models might be influenced not only by the level of estrogen, but also the imbalanced sex hormones in our study. Another explanation for the weak effect of Ar^+/−^ mice on depressive behavior is the duration of estrogen deficiency. As the reduction of endogenous estrogen in aged Ar^+/−^ female mice (12 months) was sufficient enough to promote early development of Alzheimer’s disease pathology in APP mice as we previously published (Yue et al., 2005), the less response to the estrogen deficiency-related depressive-like behavior in FST in Ar^+/−^ mice might be related to the mice at younger age (3 months) and shorter duration of estrogen deficiency (Estrada-Camarena et al., 2011). Together, although females showed higher risk of depression in human clinical studies, our data suggested that brain estrogen is important in suppressing depressive-like behaviors in both males and females in animal models.

Then we examined the estrogen-dependency in response to the dose of 10 mg/kg sertraline treatment between male and female mice. We selected the lowest effective dosage of sertraline (10 mg/kg) as our sertraline administration based on the dose-dependent response curve as shown in the Supplementary Figure S2. As shown in Figure 1, all mice responded to sertraline treatments by significant reduction of immobility time in the FST, except male Ar^+/−^ mice. The effect of sertraline administration induced a genotype-specific effect on immobility time. There was a significant less immobility time in both male and female Thy1-Ar mice, and a significant more immobility time were found in Ar^+/−^ female mice compared to sertraline-treated WT mice. Consistent with human studies in women, postmenopausal women with low level of estrogen showed worse response to SRIs treatment than premenopausal women (Pinto-Meza et al., 2006). In addition, clinical studies also showed that the combination of hormone replacement therapy (HRT) with fluoxetine (an SRI), was more effective in depression treatment than HRT (Liu et al., 2004) or fluoxetine alone (Yu et al., 2004) in post-menopausal women. The age- and estrogen-related responses to antidepressants are also reported in different clinical studies. For example, MDD patients at reproductive age were associated with a better response to SRIs while older MDD patients were associated with a superior TCAs response (Parker et al., 2003). Moreover, female MDD patients at younger age (<44 years) had significant higher rates of remission after treatment of SRIs than older (>50 years) MDD patients (Grigoriadis et al., 2003). These findings partially supported estrogen-dependent effect on antidepressants. In the human studies, antidepressants need to evoke adaptive changes after 2–4 weeks of application. However, in animal studies, acute and chronic treatment with SRIs may lead to different behavioral and neurochemical changes (Burghardt and Bauer, 2013), which offer us a clear mechanistic insight into the antidepressive actions of drugs. In our present animal study, we try to focus on the impact of estrogen levels on the acute anti-depressive effect of SRIs. We will extend our study in the future to investigate the clinical effect of sertraline in our animal models. In our current study, we showed that sertraline induced the greatest reduction of depressive-like behaviors in the Thy1-Ar mice (∼57.5% in females, ∼35.7% in males) compared to the vehicle treatment, while Ar^+/−^ mice showed the least response to the sertraline treatment (∼32.6% in females, 16.8% in males) as shown in Figure 1. Our results not only supported the estrogen-dependent effect on depression as reported by others, but also suggested that enriched brain estrogen benefits the antidepressive effect of sertraline and estrogen deficiency reduces the antidepressive effect of sertraline in our animal model. While the results from scientific experiments answer some questions, it often left new puzzles for further investigations. For example, it is not clear why male Ar^+/−^ mice did not respond significantly to sertraline in our study (Figure 1). It is possible that knocking out aromatase may also increase basal testosterone levels in males not in females, which may have affected the outcome (Amano et al., 2017). Such a sex-specific effect of the Ar^+/−^ animal model in cognitive behaviors has been reported in our previous publications as only male Ar^+/−^ mice express higher level of testosterone than age- and sex-matched WT controls and showed neuroprotective effect on Alzheimer’s related cognitive impairment as well as Alzheimer’s pathology at age of 12 months (McAllister et al., 2010), while female Ar^+/−^ mice developed early Alzheimer’s brain pathology at same age (Yue et al., 2005). Instead, females Ar^+/−^ mice are more vulnerable to the reduction of estrogen level which overrides the potential effect (if there is one) of testosterone on behavior and brain pathology. Interestingly, the estrogen-dependent influence in depressive-like behavior is not caused by potential changes of spontaneous locomotive activities in the mice. As shown in Figure 2, all three genotypes of mice showed the similar travel distance in the open field test regardless sexes.

To further understand the neuronal mechanisms of the sex difference in depression, we examined the 5-HT and DA metabolism in two brain regions in our animals. We included the PFC and HPC as the targeted brain regions to study sertraline antidepressant effect (Arnsten, 2009; Brezun and Daszuta, 2015). As showed in Figure 3, a significant elevation of 5-HIAA level and 5-HT turnover rate was found in female Ar^+/−^ mice compared to the sex-matched WT mice (Figure 3A), while higher 5-HT level and lower 5-HT turnover rate were observed in the HPC of Thy1-Ar females (Figure 3B). No changes of 5-HT or its metabolite were identified in male mice regardless genotypes and brain areas, except a reduction of 5-HT index was observed in the PFC of Thy1-Ar male mice (Figures 3C,D). Our data on the estrogen-dependent regulation of 5-HT metabolisms were consistent with previous reports from other groups, such as higher level of endogenous estrogen was associated with elevation in the level of 5-HT, and lower 5-HT turnover rate in the PFC of female mice (Kiss et al., 2012). However, estrogen injection was associated with a decrease of 5-HT levels and an increased 5-HT turnover rate (Pandaranandaka et al., 2006), or showed no differences in OVX female rat (Lu et al., 1998). It is worth to note that most of the estrogen-dependent changes of 5-HT-related depressive behaviors in animals were associated with estrogen administration acutely or chronically (Shah and Frazer, 2014) which might be different from endogenous estrogen in related to the serotonergic system (Pandaranandaka et al., 2009). Instead of treating animals with exogenous estrogen, studies of 5-HT metabolism in female animals at young, middle and old ages demonstrated that young female animals exhibited higher hippocampal 5-HT concentration than middle-age animals (Kiss et al., 2012), and women over 60 years of age had less platelet 5-HT content than younger age (Guicheney, 1988). Together, our data were in line with other reports and suggested that higher endogenous estrogen levels (younger age) might be associated with higher level of 5-HT and vice versa. Our animal models presented a unique endogenous estrogen deficiency or overexpression system for investigating the relationship between endogenous estrogen and depression. These results, combined with our behavioral data which showed reduced depression-like symptoms related to overexpression of brain estrogen and vice versa, suggested that the 5-HT system may be modulated by endogenous brain estrogen, independent of gender. Therefore, our data provided first line neurochemistry evidence of the linkage between estrogen and depressive behaviors.

As sertraline is a SRI, we extended our investigation of sex difference in the response to sertraline to brain regional changes of 5-HT and its metabolites in our animals. As shown in Figure 4, sertraline caused an increase in 5-HT and 5-HIAA levels in both brain regions of all the female mice regardless genotypes (Figures 4A,B). However, sertraline in males showed brain regional effects, such as sertraline induced greater increase of 5-HT and 5-HIAA levels in HPC than that in the PFC, while much significant reduction of 5-HIAA/5-HT ratio was found in the PFC than in the HPC compared to vehicle treated male group (Figures 4C,D). Male Thy1-Ar mice responded better to sertraline than male WT mice in terms of 5-HT and DA level changes, particularly a significant reduction of 5-HT index in the PFC from Thy1-Ar males compared to sex-matched WT males (Figure 4C). These changes suggested that sertraline induced sex-specific effect on DA and 5-HT metabolisms in the PFC and HPC and enriched brain estrogen only associated with the reduction of 5-HT index in males. However, in our experiments, the absence of the levels of 5-HT after treatment with sertraline in the PFC of male mice seems contradictory with some previous works. For example, studies of male rats demonstrated a time-dependent curve of SRI (fluoxetine) treatment on PFC monoamine levels and showed an elevation of 5-HT was started 1 h and reached to the peak at 2.5 h after the single injection of fluoxetine (Zocchi et al., 2003; Beyera, 2008). In our experiment, mice were terminated less than 1 h (∼40 min) after the sertraline injection which might be too early to observe the significant change of 5-HT levels in the PFC. Other possibilities include a variation of regional response to SRIs treatment in the PFC (the medial PFC is more sensitive than lateral PFC to SRIs-induced 5-HT) and regional 5-HT1A autoreceptors feedback mechanisms (Beyera, 2008). While we examined the PFC as a whole, the regional-specific response could be bleached out which might be partially responsible for the absence change of 5-HT induced by sertraline.

A variety of potential mechanisms have been proposed to explain the effect of estrogen levels on depression-like behaviors. First, estrogen may ameliorate the dysfunctional serotonergic activity contributing to depression-like behavior. For example, estrogen increased the mRNA expression of 5-HT synthesis key enzyme – tryptophan hydroxylase 2 (TPH2) in rat midbrain raphe nucleus (Hiroi et al., 2006). And estrogen could also upregulate the expression of 5-HT2A receptor (Sumner et al., 1999) and downregulate the expression of 5-HT1A and 5-HT1B autoreceptor in female rats (Osterlund and Hurd, 1998; Hiroi and Neumaier, 2009) to exert antidepressant effect. Besides, estrogen exhibits antidepressive action in part through neurotrophic factors and anti-inflammatory actions. While it is known that MDD often expresses a reduction of brain-derived neurotrophic factor (BDNF), study reported that estrogen treatment can increase BDNF level through binding to estrogen response element which is located on the BDNF gene (Franklin and Perrot-Sinal, 2006). Our unpublished data showed that both female and male Thy1-Ar mice showed significant increase of BDNF protein expression in HPC compared to WT. In addition, estrogens could downregulate pro-inflammatory cytokines, such as interferon gamma (IFN-γ) and interleukin-6 (IL-6) in MDD through the transcription factor NF-κB (Pozzi et al., 2006; Xu et al., 2015).

Changes of 5-HT metabolisms in specific brain regions have been also proposed in depression and response to antidepressants. Decreased ratio of 5-HIAA/5-HT in the PFC was essential for the improvement of depressive symptoms of rats (McNamara et al., 2010). Studies showed that the 5-HT index in the PFC was elevated by negative emotions (Park et al., 2012) and the 5-HT level in the HPC is known to be negatively correlation with depression-like behavior and positively correlation with antidepressant treatment (Mahar et al., 2014). In line with many published findings, estrogen appeared to facilitate the 5-HT action of SRIs and reinforced the antidepressant of SRIs via increasing 5-HT in synaptic. While short-term administration of sertraline played an essential role in 5-HT conversion in various brain regions (Muneoka et al., 2009), estrogen acted on estrogen receptor and regulated the 5-HT metabolisms indirectly through the 5-HT1A receptor (Li et al., 2013). Furthermore, estrogen can also alter the effects of SRIs on serotonin clearance through the MAPK/ERK1/2 and PI3K/Akt pathways (Benmansour et al., 2014). The metabolism of sertraline was controlled extensively by CYP 2D6 and 2C19, especially for CYP 2C19 (Probst-Schendzielorz et al., 2015). In other words, poor activity in CYP 2D6 and CYP 2C19 might elevate concentrations of sertraline, while estrogen reduced the biological function of CYP 2D6 and CYP 2C19 were suggested (Mwinyi et al., 2010; Pan and Jeong, 2015).

Several potential factors might contribute to the relationship between sex hormone and antidepressant effect in male Ar^+/−^ mice. As we mentioned before, in male Ar^+/−^ mice, in addition to reduction of endogenous estrogen, there is a great increase in endogenous testosterone level. High level of testosterone combined with fluoxetine was ineffective in alleviating depressive symptoms in male rats (Martinez-Mota and Fernandez-Guasti, 2004). Testosterone injection may cause the dysfunction of serotonin system in animals (Hernandez-Rauda and Aldegunde, 2002) and chronic testosterone administration abolished the antidepressant effect of exercise which accompanied with the improvement of oxidative damage in male rat hippocampus (Joksimović et al., 2017). Clinical research also implies that neither testosterone nor testosterone supplementation to SRIs had a positive effect on adult men with MDD (Seidman et al., 2005; Pope et al., 2010). On the other hand, testosterone administration augments the antidepressive effect in hypogonadal men (Seidman and Rabkin, 1998). This collaborative effect may due to the basal low testosterone levels in hypogonadal men. In summary, endogenous estrogen alone, but not testosterone, enhances the antidepressant of sertraline. However, the elevation of testosterone level in male Ar^+/−^ mice may influence 5-HT metabolism and eliminates the effectiveness of sertraline treatment. Sertraline may have effects on dopamine reuptake in addition to serotonin reuptake inhibition (Kitaichi et al., 2010). The dopaminergic action caused by sertraline is deemed to conduce to its positive effect on depression (Dunlop and Nemeroff, 2007), particularly in animals who are receiving close to four times the highest human dose, but unlikely in humans due to the relatively low affinity to the dopamine transporter. Our data showed that male Thy1-Ar mice are much sensitive to sertraline-induced elevation of DA levels in both PFC and HPC. Whether the sex difference in response to sertraline treatment in mice were related to the sex hormones and/or different neurotransmitters remain further investigation.

## Conclusion

Our present study indicates that endogenous estrogen has both an antidepressant-like effect as well as a beneficial effect on the efficacy of SRIs in both males and females. The neurotransmitter alterations showed that these effects were mainly associated with changes of 5-HT metabolism in both PFC and HPC. Moreover, the response of 5-HT system in female mice to sertraline treatment was more pronounced than males. However, endogenous estrogen deficiency eliminated the antidepressant effect of sertraline in FST only in male mice, suggesting that there was an interaction between estrogen, testosterone and SRIs treatment.

## Author Contributions

LM designed, conducted, and analyzed the experiments. YX, WJ, YL, and XZ contributed mice and designed the experiments. GW and RL designed and conceived the experiments. All authors discussed the results and contributed to writing the manuscript.

## Conflict of Interest Statement

The authors declare that the research was conducted in the absence of any commercial or financial relationships that could be construed as a potential conflict of interest.
