# Supplementary material for: Sex Differences in Antidepressant Effect of Sertraline in Transgenic Mouse Models
Source: Front Cell Neurosci. 2019 Feb 1;13:24. doi: 10.3389/fncel.2019.00024 (PMC6369353; doi:10.3389/fncel.2019.00024)
Supplement: Supplementary file 1 [file Data_Sheet_1.PDF]

Supplementary Material

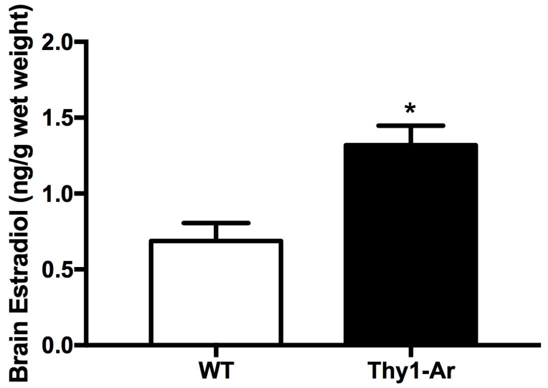


Supplementary Figure 1. The brain estradiol in WT and Thy1-Ar mice. Total estradiol was detected by enzyme-linked immunosorbent assay (ELISA) in brain from female WT (n=3) and Thy1-Ar (n=3) mice at 3 months of age. Data expressed as mean ± SEM. *P< 0.05 compared with WT mice.

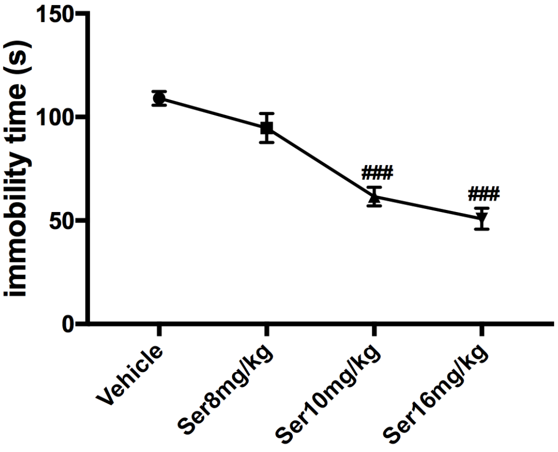


Supplementary Figure 2. Immobility time of sertraline treatment during forced swim test (FST). WT mice (n=6-8) at 2-3 months of age received single injection intraperitoneally (i.p.) of sertraline (8mg/kg, 10 mg/kg or 16 mg/kg) and vehicle. Data expressed as mean ± SEM. ###P< 0.001 compared with vehicle-treated mice.
